# Majority networks and local consensus algorithm

**DOI:** 10.1038/s41598-023-28835-2

**Published:** 2023-02-01

**Authors:** Eric Goles, Pablo Medina, Julio Santiváñez

**Affiliations:** 1 Facultad de Ingeniería y Ciencias, Universidad Adolfo Ibáñez, Avda. Diagonal las Torres 2640, Peñalolén, Santiago Chile; 2 Departamento de Ingeniería Industrial, Universidad de los Andes, Cra. 1 No. 18A 12 Bogotá, Colombia; 3 CeiBA Complexity Research Center, Carrera, 13A No. 29-24 Bogotá Colombia

**Keywords:** Mathematics and computing, Applied mathematics, Computational science, Computer science

## Abstract

In this paper, we study consensus behavior based on the local application of the majority consensus algorithm (a generalization of the majority rule) over four-connected bi-dimensional networks. In this context, we characterize theoretically every four-vicinity network in its capacity to reach consensus (every individual at the same opinion) for any initial configuration of binary opinions. Theoretically, we determine all regular grids with four neighbors in which consensus is reached and in which ones not. In addition, in those instances in which consensus is not reached, we characterize statistically the proportion of configurations that reach spurious fixed points from an ensemble of random initial configurations. Using numerical simulations, we also analyze two observables of the system to characterize the algorithm: (1) the quality of the achieved consensus, that is if it respects the initial majority of the network; and (2) the consensus time, measured as the average amount of steps to reach convergence.

## Introduction

Several new technological and social collective phenomena have emerged as a consequence of many interacting elements’ dynamics. An example of this is *consensus*, a phenomenon in which the macroscopic state of the whole system is produced when all elements of the system exhibit the same microscopic state, which may explain the emergence of the leading majority over a population of individuals. In this sense, blockchain-based applications^1–4^, dynamics of opinion formation^5–12^, physiological and ecological systems^13–15^, gene networks^16–18^, and transportation^19–21^, among others, are examples of the landscape of systems in which these majority dynamics emerge.

Even though there are various approaches to modeling these dynamics in the literature, the most common mechanisms are related to considering the local majority. Henceforth, there are two possible opinions (or states) that every individual (represented by an agent or a node in a network or cellular automata model) may assume, namely $+1$ and $-1$ in a mathematical formulation; in a resemblance to the Ising spin model of Statistical Mechanics to study magnetism^22^. Then, every individual interacts locally with individuals in his/her neighborhood, assuming the most common opinion in this set, and in case of a tie, the individual keeps his/her status unchanged. This procedure is carried out by selecting one individual randomly and repeating until dynamics reach an asymptotic state. Two of the most interesting asymptotic states are those in which the whole population assumes the same state, namely the states $+1^{*}$ (all individuals have $+1$ state) and $-1^{*}$ (all individuals have $-1$ state), in our notation. We denote any of these situations as *consensus*. In addition, other fixed points different from the consensus ones may emerge, namely for this work *spurious points*, in which the two opinions appear once the asymptotic state is reached.

Beyond different variations of the evolution rules and other algorithmic details, in a nutshell, the body of research that considers the majority rule algorithm may be divided roughly into two wide categories: one in which agent connections are changing as the system evolves^23–25^, and the other that considers static networks, in which vertex’s neighborhoods remain invariant in time^6,7,26^. In a physical interpretation, the first category represents the interaction of particles in a “gas”, where individuals are colliding with others varying their interactions as the system evolves. This model has been studied experimentally in NKN’s white paper^25^, in which every individual interacts only with a fixed number of individuals chosen randomly in each simulation step. The recurrent evolution of the system under this rule guarantees the capacity to achieve consensus. As for the second category, it may be thought of as a “crystal model”, where particles interact in a fixed network, so with fixed vicinity. This approach may be interpreted as automata networks with the local majority function^27–30^. Research of these models has provided a variety of important results attained to complex topologies (i.e., Erdos–Renyi graphs, Watts–Strograts graphs, Barabasi–Albert graphs, cellular automata), which show how consensus dynamics appear in every type of network. Results related to these topologies are useful to provide an explanation based on statistical descriptors like centrality measurements probability distributions, distance descriptors, clustering domains, etc.

The classical majority rule over finite graphs and regular lattices (“crystal” model) has been extensively studied from dynamic and complex points of view. According to Goles et al.^31,32^, dynamically, the synchronous update converges to fixed points (i.e., invariant configurations under the application of the majority rule) and two-period cycles; while the asynchronous update converges only to fixed points, but not necessarily the consensus ones. This last one has also been verified for iteration over Erdos–Renyi graphs^33–36^, obtaining the same outputs, something that let us think that eliminating the regularity does not produce better consensus results.

Given this, in this paper, we introduce the *Majority Consensus Algorithm (MCA)*, which is a variation of the usual majority rule. Unlike the classical majority where the local rule is applied always over the whole vicinity of a site, in the MCA the majority rule is applied over randomly chosen subsets in the vicinity of a site; therefore, this algorithm can be understood as a generalization of the majority rule. In this work, we study MCA in regular grid-type graphs, allowing us to characterize whether or not the MCA achieves consensus in two-dimensional lattices with four-individuals neighborhoods. When there is no consensus (i.e., the system evolves to spurious fixed points), we measure the proportion of those configurations in an ensemble of initial ones characterized by a *magnetization*-type quantity (the sum of individual states in a configuration) that do not achieve the $\pm 1^{*}$ states. It is important to mention that our analysis is developed exhaustively in the framework of every two-dimensional grid with variants of the Von Neumann Neighborhood. Actually, after applying the MCA over subsets of two or more individuals in arbitrary size grids, we prove analytically in which cases it converges to consensus, as well as in which cases the system converges to spurious fixed points. It has to be pointed out that, despite the regularity and low density of the regular grids considered in our approach (every individual has only four neighbors), one may observe a consensus-type behavior like those that appear in dense complex networks^7^. We then analyze two observables of the MCA model: (1) the quality of the achieved consensus, that is if it respects the initial majority of the network (like in the Density Classification Task in Cellular Automata^37^); and (2) the consensus time, measured as the average amount of steps to reach consensus. All cases that reach perfect consensus exhibit similar quality among them; i.e., there is no significant sacrifice in classification performance among all studied grids. Regarding the consensus time, “crystal” models have slower convergence times than the “gas” ones, especially for balanced initial configurations (similar initial amount of the two opinions).

This paper is written as follows. After this brief introduction, we present some definitions, metrics and introduce the algorithm. Then, we present our theoretical and simulation results. Finally, we present a discussion and final remarks.

## Model, algorithm and metrics

### The model and opinion formation rules

**Figure 1:**
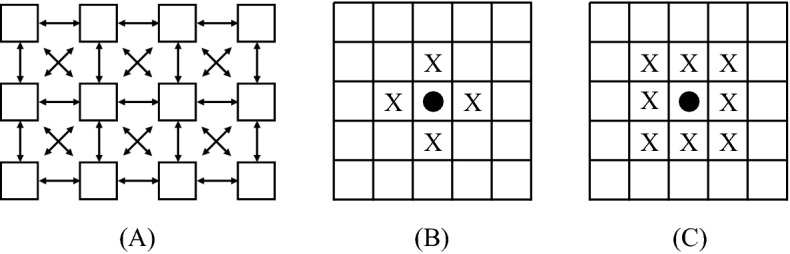
(**A**) Two-dimensional grid, (**B**) Von Neumann’s and (**C**) Moore’s Neighborhoods.

Consider a finite graph $G=(V,E)$, where *V* is the set of vertices (or individuals) and *E* is the set of edges (possible communications among individuals). Denote the set of neighbors of a vertex $v \in V$ as $N_{v}$. For the purposes of this work we use a two-dimensional $n{\times } m$ grid; i.e. a graph $G= \left( V,E \right)$ where $V=\left\{ \left( x,y \right) :1\leqslant x\leqslant n\wedge 1\le y\le m \right\}$ and $E = \{(x_1,y_1)( x_2,y_2) : |x_2 - x_1| + |y_2 - y_1| = 1\}$ (the nearest neighbor). Figure 1 shows its structure and two classical neighborhoods. Let’s define the vertex-degree *k* of a node as the number of individuals connected to this node.

**Figure 2:**
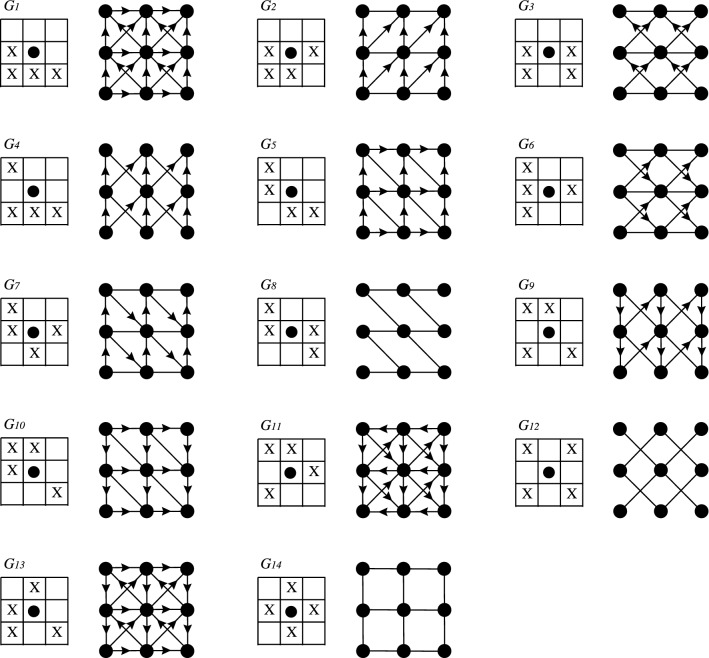
Non-equivalent (by rotations or reflections) four individuals’ vicinities and their associated $3\times 3$ grids.

Over the grid, in this study, we consider every non-equivalent four individuals’ vicinity between the eight nearest ones (as shown in Fig. 2). The reader may note that Case $G_{14}$ corresponds to the Von Neumann’s Neighborhood.

At time step *t*, each node *v* in *V* holds a state $s_v(t) \in \{-1,+1\}$, representing its opinion. As mentioned in the Introduction, the update operator is the Majority Rule over the neighborhood $N_{v}$ of vertex *v*, which is mathematically described as:

1 $$\begin{aligned} s_{v}(t+1)=f\left( s_{u}(t)\mid u \in N_{v} \right) = {\left\{ \begin{array}{ll} -1, \quad \text {if} \qquad \sum _{u \in N_v}s{_{u}(t)}< 0\\ s_{v}(t), \quad \text {if} \qquad \sum _{u \in N_v}s_{u}(t)=0\\ +1, \quad \text {if} \qquad \sum _{u \in N_v}s{_{u}(t)}> 0 \end{array}\right. } \end{aligned}$$

### The majority consensus algorithm

**Figure 3:**
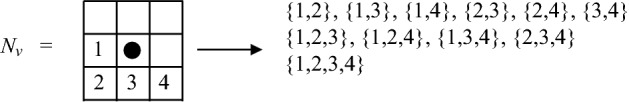
Possible selections of *k* individuals, formed by $k=$ 2, 3 or 4 nodes. At each iteration, one of the sub-neighborhoods must be selected and the *majority rule* applied.

At the beginning of the opinion dynamics each vertex is endowed with an initial state $s_v(0)$ drawn from $\{-1,+1\}$ at a certain distribution. At each time step, a node is randomly chosen and its state is updated following the Majority Consensus Algorithm (MCA): (1) randomly select subsets of $k>1$ individuals within its neighborhood (see an example in Fig. 3); and (2) apply the Majority Rule on the selected individuals.

We say that the previous procedure converges to a fixed point if and only if every state in the grid does not change under the application of the MCA. Further, we say that the procedure converges to a consensus state if and only if the fixed point is either $-1^*$ or $+1^*$.

**Figure 4:**
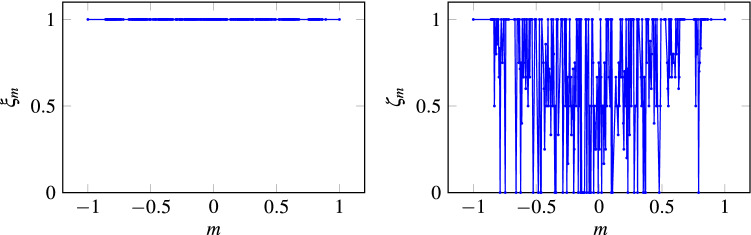
Consensus efficacy $\xi _m$ and classification efficacy $\zeta _m$ of the evolution of a set of initial configurations characterized by a magnetization *m* considering the Von Newmann vicinity, from which at each step only one neighbor is chosen and the majority rule applied. This result was obtained for a grid of $N=441$ nodes with asynchronous iterations. The reader may note that even if all configurations reach consensus ($\xi _m=1$), this approach has poor quality regarding its classification metric, i.e. $\zeta _m\ne 1$ for all instances.

**Figure 5:**
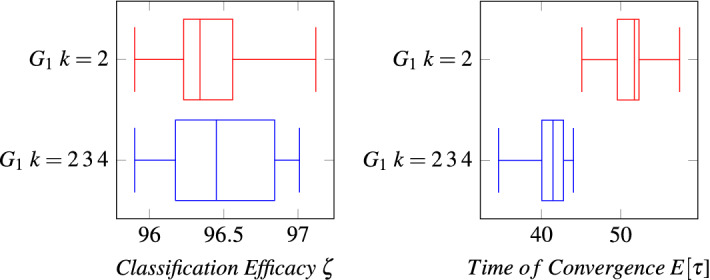
Comparison among MCA over Grid $G_1$ using $k= 2,\, 3$ or 4 neighbors and MCA using just $k= 2$. Both algorithms achieve perfect consensus and their classification efficacies do not present a significant statistical difference; the reader may note the latter in the left figure. However, MCA using $k= 2,\,3$ or 4 obtains statistically faster convergence times (right figure).

It has to be mentioned that the one-neighbor choice is intentionally forbidden. This is done to disconnect our algorithm from the Voter Model^38^ which has been already studied and proved to have poor quality consensus regarding its respect to initial majority and time of convergence, (see Fig. 4). Moreover, it has to be said that MCA includes the possibility to choose $k=$ 2, 3 or 4 neighbors for the algorithm to obtain faster times of convergence (see Fig. 5 for a comparison example among MCA using $k=$ 2, 3 or 4 neighbors and MCA using just $k=$ 2).

### Numerical simulations’ metrics

For numerical simulations, we compute the following metrics: *consensus efficacy*, *classification efficacy*, and *convergence time*. All of these metrics are defined as functions of the initial magnetization $m = (N_{+1} - N_{-1})/N$, where $N_{+1}$ and $N_{-1}$ are the number of $+1$ and $-1$ individuals respectively. Note that magnetization in our context is an operator over a state. In this sense, $m\in \{-1,+1\}$, such that when all nodes in the network have state $s_{v}=-1$ (i.e., for all *v*), $m=-1$, and when all nodes in the network have state $s_{v}=+1$, $m=+1$. To describe the three metrics mathematically, let’s define the ensemble $S^{m}(0)$ a set of $\mathbf{\vec{s}}(0)$ configurations with initial magnetization *m*. Then, we define the following subsets:

1. $S_{+1}^{m}(\infty )$ the set of the configurations that evolve from $S^{m}(0)$ and converge to $\mathbf{\vec{s}}_{\infty }=+1$.
2. $S_{-1}^{m}(\infty )$ the set of the configurations that evolve from $S^{m}(0)$ and converge to $\mathbf{\vec{s}}_{\infty }=-1$.
3. $S_{*}^{m}(\infty )$ the set of the configurations that evolve from $S^{m}(0)$ and converge to any other attractor different to $\mathbf{\vec{s}}_{\infty }=\pm 1$.
4. $S_{>0}^{m}(\infty )$ the set of the configurations that evolve from $S^{m}(0)$ and converge to a configuration which magnetization is greater than zero.
5. $S_{<0}^{m}(\infty )$ the set of the configurations that evolve from $S^{m}(0)$ and converge to a configuration which magnetization is less than zero.

Let $||\cdot ||$ be the cardinality of a set. Then, we define the *consensus efficacy* $\xi$ as:

2 $$\begin{aligned} \xi _m=\frac{||S_{+1}^{m}(\infty )||+||S_{-1}^{m}(\infty )||}{||S^{m}(0)||} \end{aligned}$$

and the *classification efficacy* $\zeta$ as:

3 $$\begin{aligned} \zeta _{m}={\left\{ \begin{array}{ll} \frac{||S_{>0}^{m}(\infty )||}{||S^{m}(0)||}, &{} \text {if} m>0\\ \frac{||S_{<0}^{m}(\infty )||}{||S^{m}(0)||}, &{} \text {if} m<0. \end{array}\right. } \end{aligned}$$

We take the expected amount of time steps for convergence of *Sm*(0) as the *convergence time*.

## Four-vicinity two-dimensional grids and consensus

**Proposition.** *From the consensus point of view, by considering any regular grid associated with four individuals of Fig.* 2, *we have the following results:*

- *For arbitrary size grids* $G_1$, $G_2$, $G_3$, $G_5$, $G_{11}$ *and* $G_{13}$ *the application of MCA converges always to consensus.*
- *For arbitrary size grids* $G_4$, $G_6$, $G_7$, $G_8$, $G_9$, $G_{10}$, $G_{12}$ *and* $G_{14}$ *there is not consensus. It means that there exist spurious fixed points different from* $-1^*$ *or* $+1^*$.

**Proof.** We must prove that, for any initial configuration of opinions considered, the application of the MCA algorithm reaches one of only two fixed points $-1^*$ or $+1^*$.

**Figure 6:**
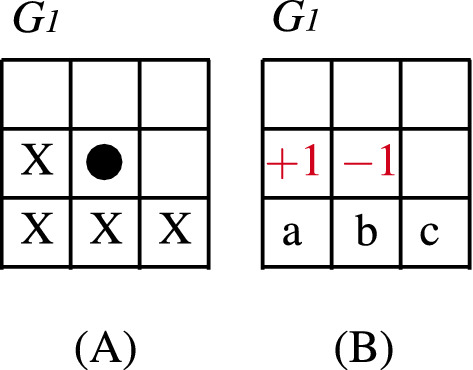
(**A**) Grid $G_1$’s vicinity. (**B**) Supposed stationary state other than $-1^*$ or $+1^*$.

Let us consider a finite grid of arbitrary size defined by the neighborhood $G_1$ (refer to Supplementary Information Annex III online for other cases, for the rest the proof is similar) and let us suppose that there is a fixed point other than $-1^*$ or $+1^*$, i.e., a stationary state in which there are opinions $-1$ and $+1$. Then, there must necessarily exist somewhere in the grid two different opinions in the same neighborhood, like its shown in Fig. 6. Since it is a fixed point then, by application of MCA for every two sites, the $-1$ has to be invariant. So, if $a={+}1$ or $b={+}1$ or $c={+}1$, then there will be two $+1s$ in the vicinity; hence, by applying the MCA over these two individuals the $-1$ will change to $+1$ which is a contradiction since we supposed the configuration was a fixed point. Now consider $a=b=c=-1$. In this case *a* and *b* are in the $+1$ neighborhood and clearly, by applying the MCA over these two individuals, the $+1$ will change to $-1$; which is a contradiction. For other initial positions of the opinion $+1$ in the neighborhood, the demonstration is the same. Therefore, the only fixed points are those of consensus. We have thus proved that, apart from the two consensus configurations, all the others are not stationary, i.e., they vary by applying the algorithm.

On the other hand, given an arbitrary configuration, there is always a way to apply the algorithm that leads to a fixed point of consensus. For example, apply the procedure to all nodes in state $+1$ until some subset is fixed, then, as the remaining $-1$ opinions cannot be fixed (there would be a spurious fixed point) there will necessarily be a choice of neighbors that carry them to opinion $+1$. Due to the monotony of the majority rule, we obtain, in this case, the convergence to the consensus state $+1^*$. The same can also be done starting by applying the procedure to the nodes in state $-1$. The important thing is that every configuration will at some point reach one of the consensus states.

**Figure 7:**
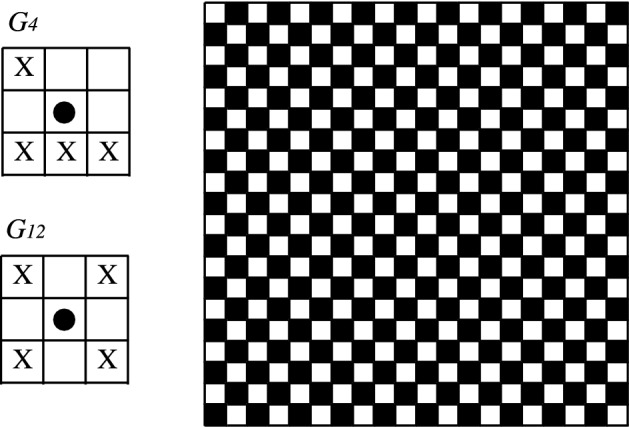
Chess configuration: spurious fixed point for grids $G_4$ and $G_{12}$. State $+1$ is represented by color black and $-1$ is represented by color white.

**Figure 8:**
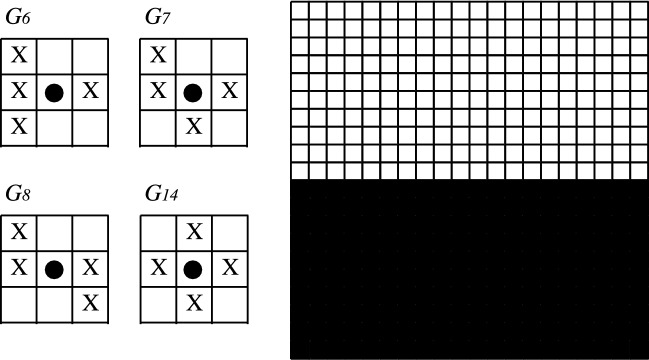
Upper/right border configuration: spurious fixed point for grids $G_6$, $G_7$, $G_8$ and $G_{14}$. State $+1$ is represented by color black and $-1$ is represented by color white.

**Figure 9:**
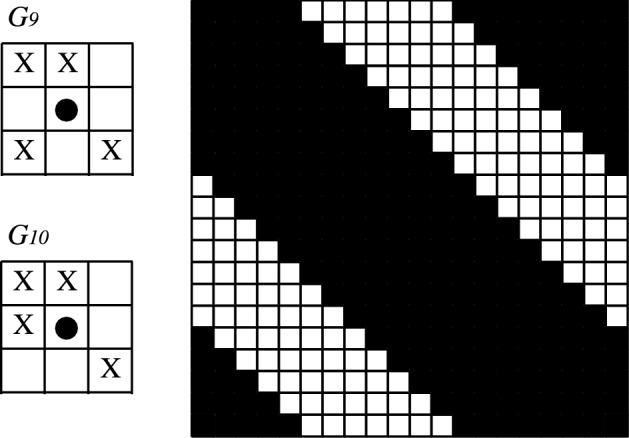
Diagonal border: spurious fixed point for grids $G_9$ and $G_{10}$. State $+1$ is represented by color black and $-1$ is represented by color white.

For grids with spurious fixed points we exhibit the following configurations: for cases $G_4$ and $G_{12}$, the chess configuration is invariant for the MCA algorithm (Fig. 7). For cases $G_6$, $G_7$, $G_8$ and $G_{14}$, the upper/right border is invariant for the MCA algorithm (Fig. 8). Finally, for cases $G_9$ and $G_{10}$, the diagonal border is invariant for the MCA algorithm (Fig. 9).

## Numerical experiments

We study the evolution of the MCA algorithm for all grids of Fig. 2. We perform simulations over $21\times 21$ toruses with an ensemble of initial configurations binomially distributed in terms of magnetizations. For each simulation, we compute the following observables: Consensus Efficacy, Classification Efficacy and Convergence Time; all as a function of the initial magnetization *m* (see definitions in a previous section).

**Figure 10:**
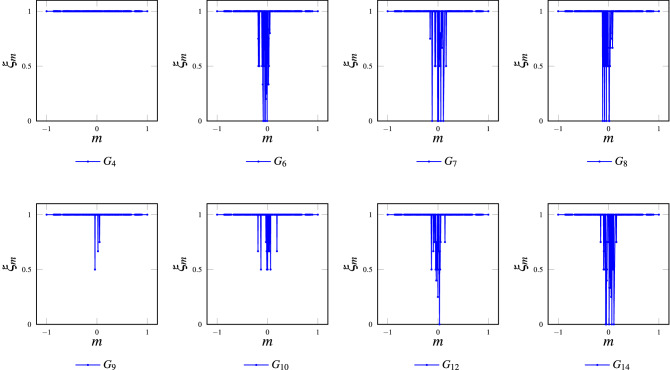
Non-perfect consensus efficacy: grids $G_4$, $G_6$, $G_7$, $G_8$, $G_9$, $G_{10}$, $G_{12}$ and $G_{14}$; $21\times 21$ Toruses, Periodic Boundary Conditions and Asynchronous Update. Grid $G_4$ has a special condition because the pattern of its spurious fixed point (chessboard) cannot be obtained from an odd number of nodes (as in a $21\times 21$ torus).

Numerical results show that, as expected, grids $G_1$, $G_2$, $G_3$, $G_5$, $G_{11}$ and $G_{13}$ achieve 100% consensus. On the other hand, Fig.  10 shows the consensus efficacy metric for grids $G_4$, $G_6$, $G_7$, $G_8$, $G_9$, $G_{10}$, $G_{12}$ and $G_{14}$; where it can be noticed that these models can not guarantee perfect consensus. Grid $G_4$ is a special case because, although it has the chessboard pattern as a spurious fixed point when the grid has an odd number of nodes (as in a $21\times 21$ torus), this pattern (chessboard) can not be obtained under periodic boundary conditions. For all models with non-perfect consensus, simulation results show a bounded range of magnetizations *m* with consensus troubles (for $m>0.2$ or $m<-0.2$ this metric has perfect behavior).

Previous results are consistent with the percentage of Initial Configurations that converge to spurious fixed points for each model (see Table 1). As it can be observed, grid $G_{12}$ shows the worst consensus performance. This behaviour is explained by the fact that this particular grid can be decomposed into 2 independent Von Neumann neighborhood sub-systems (which form a non-connected graph), so its attractors are compositions of the latter.

**Figure 11:**
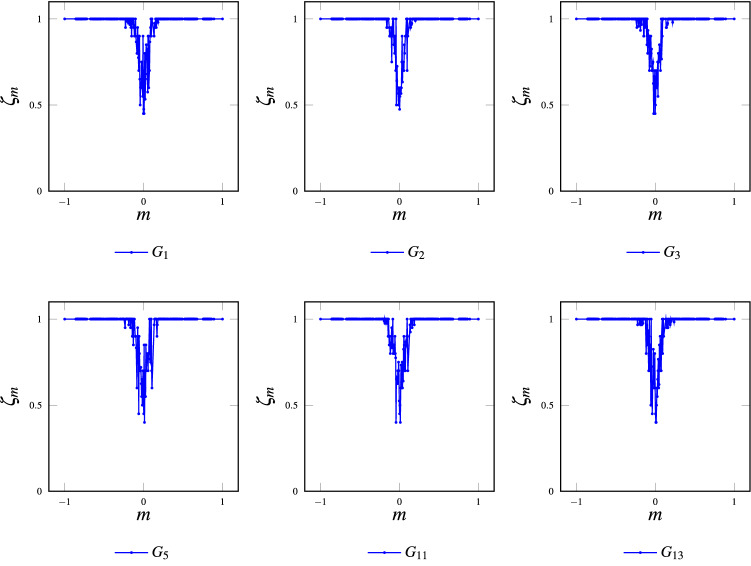
Classification efficacy: grids $G_1$, $G_2$, $G_3$, $G_5$, $G_{11}$ and $G_{13}$; $21\times 21$ toruses, periodic boundary conditions and asynchronous update.

**Figure 12:**
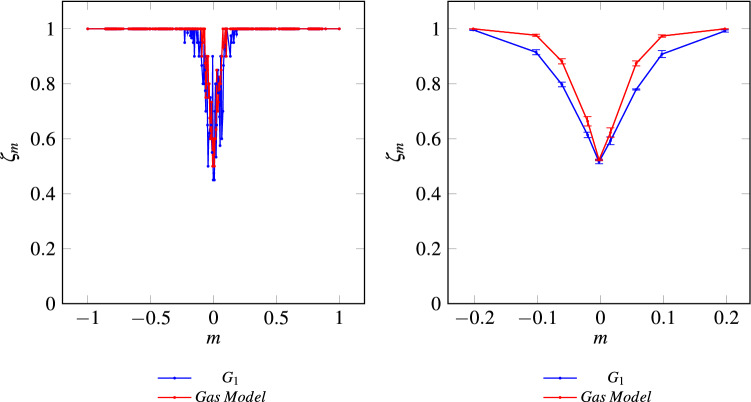
Classification Efficacy, $21\times 21$ Toruses, Grid $G_1$ and Gas model $k=2$, 3, 4. On the left, the classification efficacy over the entire range of possible magnetizations. On the right, a point-by-point estimation using $10^{3}$ initial configurations for each exact value of *m* over a smaller range $[-0.2, 0.2]$. The simulation results are averaged over five independent realizations with error bars representing the standard deviation.

The quality of consensus, measured through the Classification Efficacy, is meaningful only for models $G_1$, $G_2$, $G_3$, $G_5$, $G_{11}$ and $G_{13}$ (which reach perfect consensus). Therefore, Fig. 11 shows results only for those cases. As can be observed, MCA acting over a lattice-based “crystal model” achieves similar consensus quality for all simulated cases. In effect, results show a bounded range of magnetizations *m* with classification troubles (for $m>0.2$ or $m<-0.2$ this metric has perfect behavior). Additionally, having performed a statistical analysis using 10 independent realizations for each grid over the same ensemble of Initial Configurations, the overall classification performance does not account for significant differences ($p\text {-value}=0.668$, see Supplementary Information Annex I online). It could be interesting to perform a detailed comparison of the classification efficacy between grid models and the gas one. For the latter, at each time step, an individual is randomly chosen and two, three, or four neighbors are also randomly selected for the majority rule to be applied. Through the statistical analysis, we found no significant sacrifice in classification performance when comparing all models for magnetizations $m>0.2$ or $m<-0.2$. However, for balanced initial configurations ($-0.2< m <0.2$) the gas model outperforms grid models ($p\text {-value}=0.00001$, see Fig. 12).

**Table 1:** Percentage of initial configurations that converge to spurious fixed points.

Grid	Consensus (%)	Classification (%)	E [$\tau$]	% ICs cv spurious fixed points (%)
1	100.00	97.34	27.36	0
2	100.00	97.12	30.83	0
3	100.00	97.45	31.25	0
4	100.00	97.56	20.69	0
5	100.00	98.00	31.15	0
6	97.78	95.57	23.76	2.22
7	98.12	96.56	28.36	1.88
8	96.78	94.57	41.27	3.22
9	99.56	96.78	21.16	0.44
10	97.67	96.45	28.5	2.33
11	100.00	97.34	24.4	0
12	96.34	94.46	41.09	3.66
13	100.00	97.56	21.91	0
14	97.23	95.45	42.72	2.77

**Figure 13:**
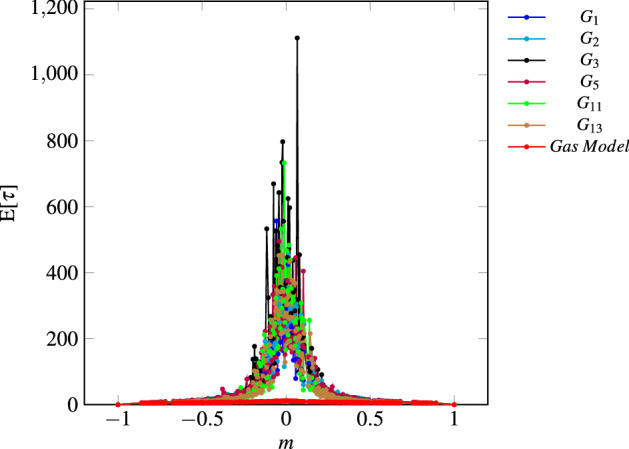
Convergence time, $21\times 21$ Toruses, Grids $G_1$, $G_2$, $G_3$, $G_5$, $G_{11}$ and $G_{13}$ and the Gas Model $k=2$, 3, 4.

As for the quality of consensus, the convergence time analysis is pertinent only for grids $G_1$, $G_2$, $G_3$, $G_5$, $G_{11}$ and $G_{13}$ (which reach perfect consensus). Therefore, Fig. 13 shows their results also compared to Gas Model for $k=2,3$ or 4. As can be observed, grid models have higher convergence times, especially for balanced magnetizations. Statistical analysis for global convergence time shows that the gas model is the fastest, and grid 3 is the slowest ($p\text {-value}=0$, please refer to Supplementary Information Annex II online, for detailed results).

## Discussion

In this paper, we introduce the application of the MCA to study consensus in two-dimensional lattices with four-individuals neighborhoods. In particular, we proved theoretically the situations in which grids of four individuals achieve or do not consensus and characterized statistically, from an ensemble of initial configurations with a particular magnetization, the proportion in which consensus is not reached. We also proposed two observables, the quality of the achieved consensus, and the consensus time to characterize the results of our algorithm.

The fact of considering a regular grid allows us to characterize in which cases consensus is reached and in which cases is not. From Fig. 2, we found that when applying MCA over subsets of two or more individuals of the grids type $G_1$, $G_2$, $G_3$, $G_5$, $G_{11}$, and $G_{13}$ consensus is always reached. On the other hand, for grids $G_4$, $G_6$, $G_7$, $G_8$, $G_9$, $G_{10}$, $G_{12}$ and $G_{14}$; there emerge spurious fixed points different from $-1^*$ or $+1^*$.

We proved that one may find consensus in low dense regular grids and we also studied, experimentally, the quality of such consensus. Our statistical results show, on average, over 96% of initial configurations regarding the initial majority (see the Density Classification Task in CA^10,20^), and all the studied grids exhibit similar quality among them (there are no significant statistical differences). In cases that perfect consensus is not reached, other attractors than $-1^{*}$ and $+1^{*}$ are always fixed points, but “spurious” ones. Experimentally we show that the attractor basin of such fixed points is small except for grid *G*12 which has a special behavior because of being formed by the composition of two independent Von Neumann sub-systems. In fact, we have proved (theoretically as well as experimentally) that small regular lattices (4-vicinity, 2-dimensional ones) may reach consensus with similar quality to gas models or dense class of networks.

Previous results could be generalized to other neighborhoods on regular grids, particularly in the two-dimensional ones studied in this work. In effect, one may consider a neighborhood other than Moore’s vicinity and inside of it select a fixed sub-neighborhood with *k* sites to build new lattices (in the paper lattices are based in 4 neighbors, as in Fig. 2). However, our characterization technique strongly depends on the regularity of the graph (number of neighbors as well as the topology) and it is not possible to extend to arbitrary families of networks. A different approach for future work, considering this same class of regular networks, is to study what happens when the local transition function, on which our MCA algorithm is based, is not necessarily the majority (which is monotone); but some other totalistic function (i.e., that depends only on the sum of the neighbors’ states). In general, it could be studied any combination of local rules to improve the quality of the MCA algorithm for some consensus network in the sense that a greater proportion of initial conditions converge to consensus in agreement with the majority opinion in the initial configuration (as in CA density problem^29,37^). Another research question is related to the robustness of the consensus algorithms. That is to study how MCA behaves with respect to local errors or attacks^25^; for instance, by considering a proportion of sites that always consider the minority opinion in its neighborhood. How will be its evolution? Does the damage (proportion of individuals with a different opinion in steady state) remain bounded or does it propagate all over the network? Another related problem is to analyze what happens when in the grid there are distrustful individuals; i.e., to change their opinion they require non the majority but rather a greater number of states in the opposite opinion (for example, a cell goes from $-1$ to $+1$ if and only if all its neighbors are in state $+1$).

## Supplementary Information

Supplementary Information.

## Data Availability

The datasets used and/or analysed during the current study are available from the corresponding author on reasonable request. Correspondence and requests for materials should be addressed to J.S.
