# Supplementary material for: Majority networks and local consensus algorithm
Source: Sci Rep. 2023 Feb 1;13:1858. doi: 10.1038/s41598-023-28835-2 (PMC9892600; doi:10.1038/s41598-023-28835-2)
Supplement: Supplementary file 1 — Supplementary Information. [file 41598_2023_28835_MOESM1_ESM.pdf]

Supplementary Information

Annex I

ANOVA Classification Results

SUMMARY

Grids	Number	Sum	Average	Variance
1	10	964,86	96,486	0,16469333
2	10	965,52	96,552	0,21739556
3	10	966,07	96,607	0,18677889
5	10	964,51	96,451	0,08703222
11	10	963,31	96,331	0,26234333
13	10	963,52	96,352	0,18550667

Supplementary Table S1. Summary of data used in the Anova Analysis - Classification Results

ANALYSIS

Variation Origin	Square Sum	Freedom Degrees	Square Averages	F	Probability	Critical Value for F
Among Grids	0,590748333	5	0,118149667	0,642263	0,668448738	2,386069862
Inside Grids	9,93375	54	0,183958333			
Total	10,52449833	59				

Supplementary Table S2. Anova Analysis - Classification Results

If the value of the F statistic is less than its critical value, the null hypothesis is accepted (there are no significant differences).

Annex II

ANOVA Convergence Time Results

SUMMARY

Grids	Number	Sum	Average	Variance
1	10	408,0144124	40,80144124	9,708154349
2	10	481,1829268	48,11829268	26,26752383
3	10	657,594235	65,7594235	98,83852778
5	10	477,7039911	47,77039911	14,13411751
11	10	496,6929047	49,66929047	30,24651224
13	10	472,6463415	47,26463415	42,37835361

Supplementary Table S3. Summary of data used in the Anova Analysis - Convergence Time Results

ANALYSIS

Variation Origin	Square Sum	Freedom Degrees	Square Averages	F	Probability	Critical Value for F
Among Grids	3490,131008	5	698,0262017	18,90191328	8,108E-11	2,38606986
Inside Grids	1994,158704	54	36,92886489			
Total	5484,289712	59				

Supplementary Table S4. Anova Analysis - Convergence Time Results

The value of the F statistic less than its critical value implies that the null hypothesis is rejected (there are significant differences). Through Tukey Method, significant differences are found for the following pairs o grids: 1 and 3; 1 and 11; 2 and 3; 3 and 5; 3 and 11; 3 and 13.

Annex III

Additional Examples - Theoretical Results

Proof. Consider grid G_2, we must prove that, for any initial configuration of opinions considered, the application of the MCA algorithm reaches one of the only two fixed points -1^* or $+1^*$.

Let us consider a finite grid of arbitrary size defined by the neighborhood G_2 (for the rest of cases the proof is similar) and let us suppose that there is a fixed point other than -1^* or $+1^*$, i.e., a stationary state in which there are opinions -1 and $+1$ (like its shown in Supplementary Figure S1). Then, there must necessarily exist somewhere in the grid two different opinions in the same neighborhood, for instance:

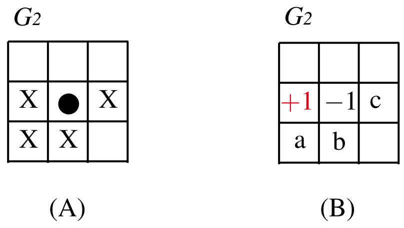

Supplementary Figure S1. (A) Grid G_2's vicinity. (B) Supposed stationary state other than -1^* or $+1^*$

Since it is a fixed point then, by application of MCA for every two sites, the -1 has to be invariant. So, if $a = +1$ or $b = +1$ or $c = +1$, then there will be two $+1s$ in the vicinity; hence, by applying the MCA over these two individuals the -1 will change to $+1$ which is a contradiction since we supposed the configuration was a fixed point. Now consider $a = b = c = -1$. In this case a and the -1 are in the $+1$ neighborhood and clearly, by applying the MCA over these two individuals, the $+1$ will change to -1; which is a contradiction. Therefore, the only fixed points are those of consensus.

Proof. Consider grid G_3, we must prove that, for any initial configuration of opinions considered, the application of the MCA algorithm reaches one of the only two fixed points -1^* or $+1^*$.

Let us consider a finite grid of arbitrary size defined by the neighborhood G_3 (for the rest of cases the proof is similar) and let us suppose that there is a fixed point other than -1^* or $+1^*$, i.e., a stationary state in which there are opinions -1 and $+1$ (like its shown in Supplementary Figure S2). Then, there must necessarily exist somewhere in the grid two different opinions in the same neighborhood, for instance:

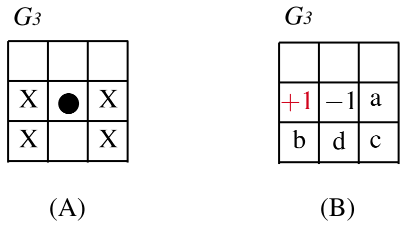

Supplementary Figure S2. (A) Grid G_3's vicinity. (B) Supposed stationary state other than -1^* or $+1^*$

Since it is a fixed point then, by application of MCA for every two sites, the -1 has to be invariant. So, if $a = +1$ or $b = +1$ or $c = +1$, then there will be two $+1s$ in the vicinity; hence, by applying the MCA over these two individuals the -1 will change to $+1$ which is a contradiction since we supposed the configuration was a fixed point. Now consider $a = b = c = -1$. In this case the -1 and d are in the $+1$ neighborhood and clearly, by applying the MCA over these two individuals, d must be $+1$ to be in a tie with the -1 and the $+1$ remain invariant. This is a contradiction because the b and c are in d's neighborhood and by choosing these two individuals d will change to -1. Therefore, the only fixed points are those of consensus.

Proof. Consider grid G_5, we must prove that, for any initial configuration of opinions considered, the application of the MCA algorithm reaches one of the only two fixed points -1^* or $+1^*$.

Let us consider a finite grid of arbitrary size defined by the neighborhood G_5 (for the rest of cases the proof is similar) and let us suppose that there is a fixed point other than -1^* or $+1^*$, i.e., a stationary state in which there are opinions -1 and $+1$ (like

its shown in Supplementary Figure S3). Then, there must necessarily exist somewhere in the grid two different opinions in the same neighborhood, for instance:

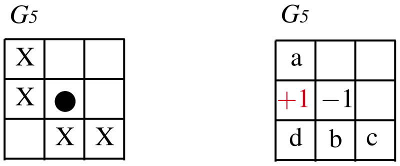

(A) (B)

Supplementary Figure S3. (A) Grid G_5's vicinity. (B) Supposed stationary state other than -1^* or $+1^*$

Since it is a fixed point then, by application of MCA for every two sites, the -1 has to be invariant. So, if $a = +1$ or $b = +1$ or $c = +1$, then there will be two $+1s$ in the vicinity; hence, by applying the MCA over these two individuals the -1 will change to $+1$ which is a contradiction since we supposed the configuration was a fixed point. Now consider $a = b = c = -1$. In this case d and b are in the $+1$ neighborhood and clearly, by applying the MCA over these two individuals, d must be $+1$ to be in a tie with b and the $+1$ remain invariant. This is a contradiction because the $+1$ and $d = +1$ are in b's neighborhood and by choosing these two individuals b will change to $+1$. Therefore, the only fixed points are those of consensus.
